# Surface roughness profile separation using singular spectrum analysis

**DOI:** 10.1371/journal.pone.0336936

**Published:** 2025-11-25

**Authors:** Ziming Pang, Xiaochuan Gan, Ming Kong

**Affiliations:** 1 College of Metrology, Testing and Instrumentation, China Jiliang University, Hangzhou, China; 2 AVIC Changcheng Institute of Metrology & MeasurementBeijing, China; IIIT Kurnool: Indian Institute of Information Technology Design and Manufacturing Kurnool, INDIA

## Abstract

Surface roughness is a critical parameter used to describe the microscopic geometric deviations of a part, and serves as an essential indicator for assessing the quality of surface processing in various mechanical components. This study evaluates Singular Spectrum Analysis (SSA) for surface roughness profile separation, comparing its effectiveness with the ISO standard Gaussian filter. Using NIST roughness measurement data, this study investigates how SSA’s window length and grouping method affect roughness parameters. The findings indicate that with an appropriately chosen window length, the SSA technique can effectively separate roughness signals and yield roughness parameter values comparable to those obtained using the Gaussian filter, such as the arithmetical mean deviation of the assessed profile (*Ra*), the root mean square deviation of the assessed profile (*Rq*), and the kurtosis of the assessed profile (*Rku*). These findings establish SSA as a viable alternative for surface roughness profile separation, with broad applications in surface metrology.

## 1. Introduction

The surface irregularities and dimensions produced during machining significantly influence the quality and performance of workpieces. This impact extends beyond its fundamental function to include tribological properties, aerodynamic and hydrodynamic performances, fatigue resistance, corrosion resistance, and other technical functions. The final surface irregularities and shape of the product are critical in determining whether the surface characteristics are advantageous or detrimental. However, unpredictable factors like tool wear and material variations complicate surface roughness formation.Consequently, the development of reliable methods for surface roughness separation is crucial for ensuring a high product performance.

In a machined surface profile, high-frequency components represent surface roughness, while medium- and low-frequency components correspond to waviness and shape errors, respectively [1]. Surface roughness is obtained by filtering the machined surface contour to remove waviness and measurement noise. The measurement signal of the machined surface encompasses shape error, waviness profile, and roughness profile. To isolate the roughness profile, the surface contour is filtered to remove waviness and shape errors.

Filtering is an essential process employed to distinguish waviness and roughness from each other and from finer, higher-frequency content such as instrument noise [2]. In the early stages of surface topography contact measurement and data processing utilizing analog circuits, filtering was accomplished using 2RC analog filters. These 2RC filters introduce distortion to the surface profile shape owing to their inherent nonlinear phase shift, which significantly affects the evaluation of profile parameters [3]. With the advancement of research and the development of computer technology, the standard issued by the International Organization for Standardization (ISO) in 1996, ISO 11562–1996, explicitly adopted the Gaussian filter median as the surface contour baseline [4]. ISO 16610–21, effective June 2011, replaced ISO 11562–1996 and defines Gaussian filter properties [5]. Gaussian filters are currently the most widely used for surface roughness analysis [5]. Gaussian filters, spline filters, and their corresponding robust filters are recommended as ISO standard contour filters [6–8].

Various digital signal-processing techniques have been employed for surface characterization, including those based on Fourier and wavelet transforms [9,10]. L. V. Markova et al. utilized empirical modal decomposition techniques for the extraction of surface roughness profiles [11]. Sergey N. Grigoriev et al. applied morphological filtering methods to assess surface texture parameters, thereby estimating the functional properties of product surfaces and achieving results comparable to those obtained with Gaussian or spline filters [12]. Yan Y. Q. et al. developed a machine vision-based method for surface roughness measurement, supporting in-process inspection [13].

Singular Spectrum Analysis (SSA) is a non-parametric method based on multivariate statistics for analyzing time series data. This study evaluates SSA’s performance in filtering machined surface contours to measure roughness. To achieve this objective, this study compares the outcomes of the SSA technique with those obtained using the ISO-specified Gaussian filter.

## 2. Singular spectrum analysis

Singular Spectrum Analysis (SSA) is a non-parametric, model-independent method for time-series analysis. SSA requires no statistical assumptions about signal or noise properties, such as smoothness or ergodicity. Thus, it is not a statistical method in the traditional sense of the term [14]. SSA originated with Broomhead and King’s work on time-series decomposition [15]. The concept of SSA was independently developed in Russia (St. Petersburg, Moscow) and by several groups in England and the United States. Subsequently, Danilov, Zhigljavsky, Golyandina, and others provided a comprehensive exposition of the theoretical and practical underpinnings of the SSA [16].

The SSA has been employed to extract trends and filter out noise from various time-series signals, including climatic and geophysical sequences [17,18]. Furthermore, SSA also identifies periodic motions in complex dynamic systems [19], relevant to surface roughness analysis. Wang [20] was the first to apply the SSA technique to analyze machine faults using vibration signals, discovering that rotor-stator friction faults in rotating machinery result in increased noise in the vibration signals compared to normal conditions. Niu et al. employed the SSA for noise reduction in LiDAR signals [21]. Currently, SSA is applied to detect tool conditions [22–24] and monitor surface roughness during machining [25].

SSA consists of two main stages: decomposition, which breaks down the signal, and reconstruction, which rebuilds it. These components are further divided into four specific tasks: embedding, singular value decomposition, grouping, and diagonal averaging.

### 2.1. Decomposition

1) Embedding

Given a one-dimensional time series of length *N* as $𝐬=[s_{1},s_{2},\cdots ,s_{N-1},s_{N}{]}^{T}\in {\mathbb{R}}^{N}$ , define an appropriate window length of *L*. This step segments a one-dimensional signal into *K* overlapping segments of length *L* using a sliding window as


$${𝐱}_{k}=[s_{k},s_{k+1},\cdots ,s_{k+L-1}{]}^{T}\in {\mathbb{R}}^{L}$$ (1)

where *s* is the value of the signal intercepted by the window, *k* = 1,2,...,*K*, and *K* = *N*-*L* + 1.

Subsequently, a trajectory matrix is constructed by


$$𝐗=\left[{𝐱}_{1},{𝐱}_{2},\cdots ,{𝐱}_{K}\right]\in {\mathbb{R}}^{L\times K}$$ (2)

It is obvious that the diagonal elements of the trajectory matrix **X** are the same; therefore, this trajectory matrix **X** is a Hankel matrix, where


$$𝐗=\left[\begin{array}{cccc} {}*20cs_{1} & s_{2} & \cdots & s_{K}\\ s_{2} & s_{3} & \cdots & s_{K+1}\\ \vdots & \vdots & \ddots & \vdots \\ s_{L} & s_{L+1} & \cdots & s_{N} \end{array}\right]$$ (3)

This embedding corresponds to the Takens embedding [26], which is used to reconstruct the system’s trajectory in a reconstructed phase space from time-series data. In SSA, the lagged vectors play the role of coordinates in this phase space, enabling the extraction of underlying dynamical components without assuming a specific parametric model.

2) Singular Value Decomposition

Consider the correlation matrix **S** = **XX**^T^ as a symmetric matrix and perform a singular value decomposition of the matrix **S**, that is,


$$𝐒=𝐗{𝐗}^{T}=𝐔\Lambda {𝐔}^{T}$$ (4)

where $\Lambda =diag(\lambda_{1},\cdots ,\lambda_{L})$.

Λ is a diagonal matrix, and the eigenvalues on the diagonal matrix are arranged in descending order. The column vectors of **U**=(**u**_**1**_, **u**_**2**_, …, **u**_***L***_) are the corresponding eigenvectors, which are mutually orthogonal. The trajectory matrix **X** can then be expressed as a sum of *L* matrix components of rank 1:


$$𝐗=\sum_{i=1}^{L}\sqrt{\lambda_{i}}{𝐮}_{i}{𝐯}_{i}^{T}={\tilde{𝐗}}_{1}+\cdots +{\tilde{𝐗}}_{L}$$ (5)

where ${𝐯}_{i}=\frac{{𝐗}^{T}{𝐮}_{i}}{\sqrt{\lambda_{i}}},\;i=1,\cdots ,L$, $\tilde{X}$, …, ${\tilde{X}}_{L}$, are the *L* matrix components.

It can be seen that $\sqrt{\lambda_{i}}$ is the singular value of the trajectory matrix, while the column vectors of **U**=(**u**_**1**_,**u**_**2**_,…,**u**_**L**_) and **V**=(**v**_**1**_,**v**_**2**_,…,**v**_**L**_) are the left singular value vector and the right singular value vector corresponding to the singular value $\sqrt{\lambda_{i}}$ (*i = 1*,..., *L*) about the trajectory matrix, respectively.

### 2.2. Reconfiguration

1) Grouping

In accordance with the established rules and numerical guidelines, *L* matrices X_i_ with rank 1 are organized and integrated into several distinct groups, with the matrices within each group subsequently summed. If index set *J*={1,2,…,*L*} is partitioned into disjoint subsets *I*_1_,*I*_2_,…,*I*_M_, then each corresponding subset can be expressed as


$${\hat{X}}_{p}=\sum_{j\in I_{p}}{𝐗}_{j}$$ (6)

where *p* = 1,2,…,*M*.

Each group’s summed matrix retains the *L* × *K* dimensions of the original trajectory matrix, enabling complete reconstruction, specifically,


$$𝐗=\sum_{p=1}^{M}{\hat{X}}_{p}$$ (7)

2) Diagonal averaging

This step converts each grouped matrix ${\hat{X}}_{p}(p=1,2,\cdots ,M)$ into a one-dimensional time series, termed SSA components.

Let ${\hat{x}}_{t,p}$ denote the *t*-th element of the *p*-th SSA component and ${\bar{x}}_{a,b,p}$ the element at row *a* and column *b* of matrix X. The diagonal averaging formula is as follows:


$${\hat{x}}_{t,p}=\{\begin{array}{cc} {}*20l\frac{\text{1}}{t}\sum_{m=1}^{t}{\bar{x}}_{m,t-m+1,p} & for\;1\leq t<L\\ \frac{\text{1}}{L}\sum_{m=1}^{L}{\bar{x}}_{m,t-m+1,p} & for\;L\leq t<K\\ \frac{\text{1}}{N-t+1}\sum_{m=t-K+1}^{L}{\bar{x}}_{m,t-m+1,p} & for\;K\leq t\leq N \end{array}$$ (8)

where, *t* = 1,…,*N* and *p* = 1,…,*M*.

In the computation process, the diagonal sum is equivalent to the convolution of *u*_*i*_ and *v*_*i*_. Therefore, by utilizing the FFT algorithm, the computational complexity can be significantly reduced, greatly improving the efficiency of the SSA method.

## 3. Difficulties in the application of SSA

SSA involves four steps—embedding, singular value decomposition, grouping, and diagonal averaging—as described in Section 2. Notably, two key challenges require attention: selecting the window length (*L*) and determining the grouping method. These challenges are the primary focus of this study’s SSA application.

### 3.1. The determination of the window length

Selecting an appropriate window length (*L*), the number of signal samples per segment, is critical for SSA’s performance. SSA’s ability to separate roughness, waviness, and noise depends on the window length *L*. A larger *L* value results in increased computational demands and an extended processing time. The window length remains consistent for the singular-value decomposition of the trajectory matrix, whether *L* or *K* = *N* − *L* + 1. Therefore, it is imperative to select an appropriate signal length as the window length.

In SSA, the window length *L* is the key parameter, determining the precision of signal decomposition and reconstruction. As the only user-defined parameter in decomposition, *L* directly affects SSA’s ability to capture time-series dynamics and separate roughness from surface profile. However, existing studies have shown that there is no universal criterion for selecting the window length, and its optimal value needs to be dynamically adjusted according to the specific application scenarios, data characteristics, and a priori knowledge.

In general, the window length should be as large as possible. When *L* is sufficiently large, each lag vector ${𝐱}_{k}=(s_{k},s_{k+1},\cdots ,s_{k+L-1}{)}^{T}\in {\mathbb{R}}^{L}$ of *L* can be treated as a single sequence, allowing the study of the specific dynamic properties within this set of sequences. Given that most weak separations of time-series components are asymptotic in nature, selecting a relatively large window length is essential for achieving better separation in most cases. Conversely, a small L may obscure roughness and waviness components. Thus, the window length should be sufficiently large to ensure that the weak separation remains stable, even with minor perturbations in *L*.

For complex signals, a large *L* may cause aliasing, mixing roughness with waviness components. Conversely, a significant reduction in *L* may result in poor separation quality. In certain instances, minor adjustments to *L* can minimize component mixing and enhance separation, transitioning from weak to strong separation. Therefore, the window length is typically chosen to be as large as possible; however, if it is too large or too small, the separation may become ineffective.

Initially, the window length was suggested to range from 2 to the sequence length. However, in 1996, Elsner and Tsonis, through a series of discussions and arguments in their work, proposed that the window length should generally be set to one-quarter of the time-series length [27]. In 2001, Golyandina noted in a treatise on singular spectrum analysis and related techniques that excessively large or small window lengths could lead to undesirable outcomes such as over-decomposition or poor separation quality. Consequently, Golyandina recommended determining an appropriate window length through multiple experiments for practical applications [28]. For instance, in 2018, Xu et al. conducted comparative experiments with different window lengths during the denoising and extraction of EEG signals, ultimately identifying a suitable window length [29]. Similarly, in 2024, Nie determined an appropriate window length through repeated simulation comparisons [30]. Although the selection of the window length typically requires confinement within a reasonable range, no universally applicable criterion has been established yet. In practice, it is often necessary to conduct iterative experiments tailored to a specific time series, comparing decomposition outcomes across different dimensions, to determine the optimal value. However, as this approach depends heavily on the characteristics of the specific data, its findings cannot be generalized to other contexts. Therefore, the scientific and efficient selection of the window length remains a critical theoretical issue in the field of singular spectrum analysis, which urgently requires resolution

### 3.2. The grouping method

In the earliest proposed method for grouping in SSA, the components were categorized into two groups. The process begins by sorting all eigenvalues from largest to smallest, followed by sequential accumulation starting with the first eigenvalue. When this cumulative value reaches or surpasses the product of the total sum of all the eigenvalues and a predetermined threshold, the eigenvalues are divided into two distinct groups. However, the success of this method relies heavily on the appropriateness of the chosen threshold, which is crucial for the final outcome of the SSA. For instance, in 2018, Lin introduced a method for determining grouping based on an empirical pattern decomposition technique [31]; in 2024, Liu proposed a method for grouping based on correlation coefficients [32].

Following the singular value decomposition of the trajectory matrix, a series of singular values and their corresponding left and right singular vectors can be obtained. Typically, the right singular vector is referred to as the principal component, which indicates the trend of its associated components. By leveraging this characteristic, an unsupervised grouping strategy can be developed by examining the power spectral density or various entropy value metrics of the principal components. This method facilitates an automated grouping process, thereby minimizing the influence of human factors on results. For instance, in 2024, Huang grouped SSA components into trend and periodic terms [33]. By integrating this with the common mode error (CME), the method achieves grouping.

Another approach is to first transform the rank-1 biorthogonal matrices into one-dimensional singular spectral components through diagonal averaging, and then group these components. A distance-based grouping method involves the following steps to cluster SSA components. First, similarity between SSA components is assessed using metrics like weighted correlation. Second, a proximity matrix is constructed based on these distances. Finally, a distance-based clustering algorithm, such as hierarchical clustering, is employed to automatically group the one-dimensional components. Although the process of this method is intuitive, its core lies in selecting appropriate clustering algorithms to ensure the accuracy and reasonableness of the grouping results. For example, Bilancia et al. used all-connected hierarchical clustering, though its rationale is unclear [34]. Further studies have explored distance-based grouping methods for SSA. Kalantari et al. introduced a new difference measure based on multiple matrix paradigms to quantify the difference between two one-dimensional components [35]. They subsequently optimized the grouping strategy by comparing the performance of different hierarchical clustering structures in singular spectrum grouping. The results indicate that the method enables faster and more accurate grouping, thereby enhancing the accuracy of the reconstructed sequences and prediction results. The literature [36] suggests using a neural network optimized by the firefly algorithm to group the components according to the magnitude of their contribution.

## 4. Experimental Verification

**Fig 1:**
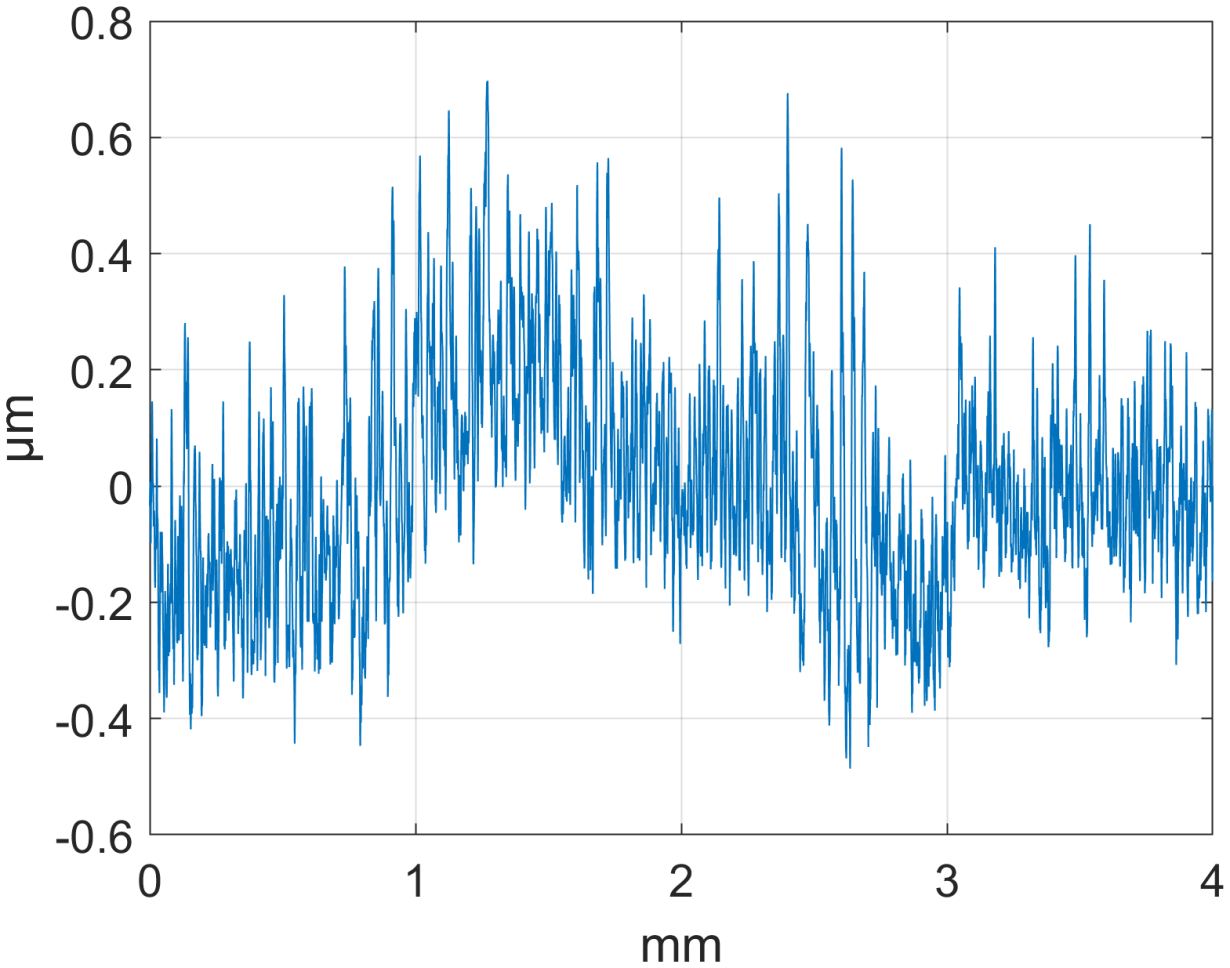
Original Outline: d_fine.

**Fig 2:**
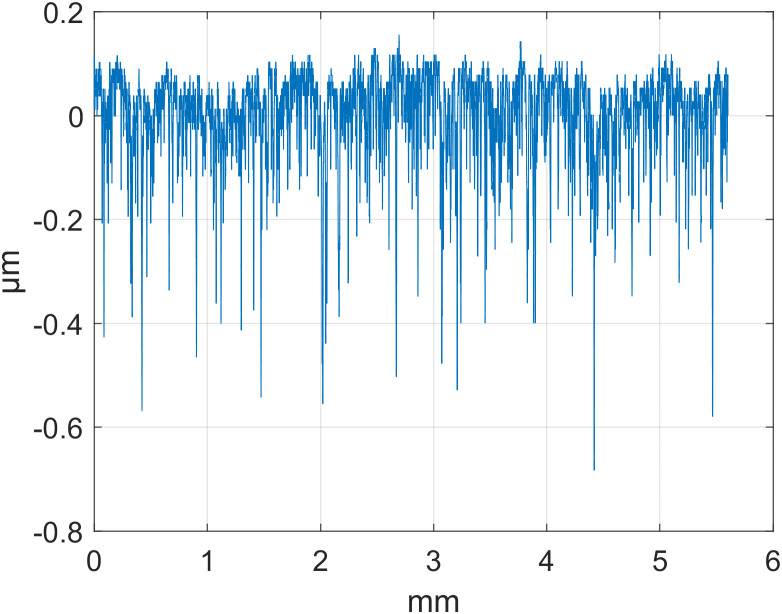
Original Outline: Polish.

**Fig 3:**
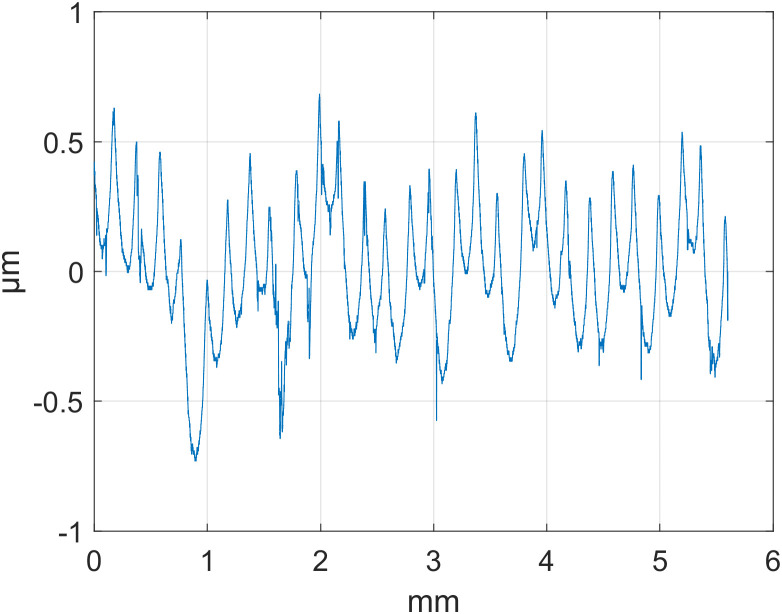
Original Outline: Mills.

To evaluate the effectiveness of the SSA technique in separating roughness signals, an experiment was conducted using surface roughness measurement data provided by National Institute of Standards and Technology (NIST). We selected surface profile data for three parts—d_fine (finely machined), Polish (polished), and Mill (milled)—for analysis. All of them used a stylus roughness measuring instrument with a sampling interval of 0.5 µm and a sampling point of T = 8000. The detailed data are presented in Table 1, and the original contours are shown in Figs 1–3.

**Table 1:** Detailed data of parts.

Part name	Sampling interval	Sampling point (*T*)
d_fine	0.5 µm	8000
Polish	0.25 µm	22401
Mill	0.25 µm	22401

In this test, the parameters of the Gaussian filter are configured as follows: sampling interval, 0.5 µm or 0.25 µm; sample length, 4 mm or 5.6 mm; cut-off wavelength, 0.8 mm; cut-off constant, 0.5.

### 4.1. Selection of window length

Initially, the window length (*L*) was tested by selecting *L* as 200μm, 300μm, 400μm and 500μm of sampling point (*T*) of sampling point (*T*), rounded down. Surface roughness is typically assessed using various parameters, including height, space, mixing, material ratio, volume, fractals, features, elements, and patterns. Among them, the height parameter is determined based on the contour ordinate and is subject to change during the filtering process. Therefore, to evaluate the filtering effect, we selected three parameters within the height parameters: the arithmetical mean deviation of the assessed profile (*Ra*), the root mean square deviation of the assessed profile (*Rq*), and the kurtosis of the assessed profile (*Rku*). *Ra* and *Rq* are calculated based on the average contour height, providing strong statistical robustness against individual features. *Rku* is more sensitive to the significant effects of isolated peaks or pits. Additionally, *Ra* and *Rq* are strongly correlated, reflecting similar profile characteristics. The *Rku* parameter is primarily used to assess the presence of isolated peaks and valleys. The calculation methods for *Ra*, *Rq*, and *Rku* are shown in equations 9–11 and the comparative data are shown in Table 1.


$$Ra=\frac{\text{1}}{l}\int_{0}^{l}\left|Z(x)\right|dx$$ (9)

$$Rq=\sqrt{\frac{\text{1}}{l}\int_{0}^{l}Z^{2}(x)dx}$$ (10)

$$Rku=\frac{\text{1}}{Rq^{4}}\sqrt{\frac{\text{1}}{l}\int_{0}^{l}Z^{4}(x)dx}$$ (11)

To comprehensively compare the characteristics of SSA, this study also conducted a comparison with the multiresolution analysis method of wavelet decomposition. The wavelet analysis parameters were configured as follows: the ISO-recommended Cubic B-spline wavelet was employed with 10 decomposition levels, where the highest level was assigned to the surface profile and waviness, and the sum of the remaining levels was considered as the roughness component.

**Fig 4:**
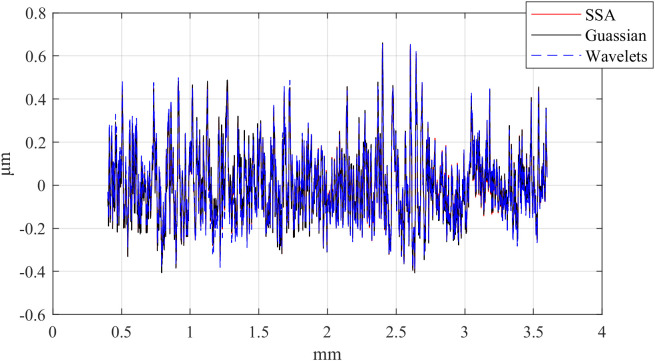
Comparison of roughness separation effect at *L* = 300μm: d_fine.

**Fig 5:**
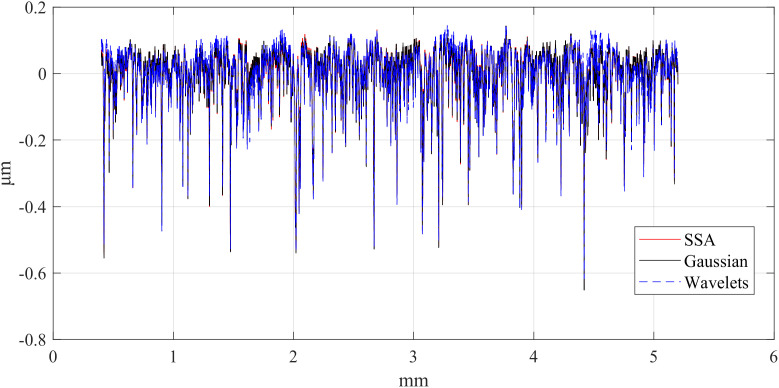
Comparison of roughness separation effect at *L* = 300μm: Polish.

**Fig 6:**
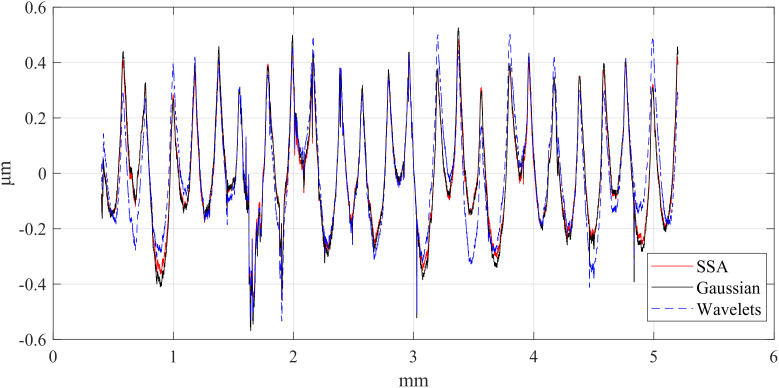
Comparison of roughness separation effect at *L* = 300μm: Mill.

First, the computation times of the three methods are compared. The computations were performed in MATLAB R2023b on a platform equipped with an i5-12450H CPU and an RTX 4060 Laptop GPU. Table 2 presents the computation times of the different methods, Table 3 compares the RMSE between Gaussian-filtered signals and SSA at different window lengths, and Table 4 lists the test data for various window lengths.Figs 4–6 shows a comparison of roughness separation effects at *L* = 300 μ m.

**Table 2:** Computation time (seconds).

Part name	SSA				小波
	*L* = 200 μm	*L* = 300 μm	*L* = 400 μm	*L* = 500 μm	Cubic b-spline
d_fine	0.1899	0.3088	0.4661	0.6548	0.0206
Polish	1.2505	2.4017	3.8953	7.0910	0.0142
Mill	1.4732	2.3619	3.8876	6.5192	0.0196

**Table 3:** — RMSE with respect to Gaussian-filtered signal (μm).

Part name	SSA				小波
	*L* = 200 μm	*L* = 300 μm	*L* = 400 μm	*L* = 500 μm	Cubic b-spline
d_fine	0.0162	0.0051	0.0041	0.0123	0.0249
Polish	0.0094	0.0040	0.0121	0.0102	0.0180
Mill	0.0558	0.0195	0.0354	0.0664	0.0796

**Table 4:** Roughness Parameters.

Part name	Roughness Parameters	NIST reference value	Gaussian filter	SSA			
				*L* = 200 μm	*L* = 300 μm	*L* = 400 μm	*L* = 500 μm
d_fine	*Ra*(µm)	0.12622	0.12622	0.12308	0.12527	0.12674	0.12782
	*Rq*(µm)	0.15896	0.16043	0.15702	0.15927	0.16101	0.16243
	*Rku*	3.3264	3.54712	3.60222	3.55471	3.53458	3.52444
Polish	*Ra*(µm)	0.06161	0.06161	0.06003	0.06091	0.06114	0.06150
	*Rq*(µm)	0.08702	0.08768	0.08579	0.08689	0.08721	0.08769
	*Rku*	10.45915	10.41407	10.28693	10.36135	10.0648	10.05247
Mill	*Ra*(µm)	0.16423	0.16424	0.14321	0.15389	0.15784	0.16339
	*Rq*(µm)	0.20113	0.20134	0.17249	0.18819	0.19395	0.20103
	*Rku*	2.40393	2.48783	2.50507	2.47912	2.65520	2.81395

The test results indicate that the computational time of SSA increases approximately linearly with the window length. Analysis of the RMSE trend shows that an excessively small window length leads to insufficient decomposition, whereas an excessively large window length causes over-decomposition; both cases reduce accuracy. Therefore, an appropriately chosen window length can improve accuracy while reducing computation time. Based on a comprehensive comparison, the optimal window length for all three workpieces was determined to be *L* = 300μm. The corresponding roughness parameters and their error ratios are summarized in Table 5.

**Table 5:** Error ratios at the optimal window length.

Part name	Roughness Parameters	NIST reference value	Gaussian filter	SSA	Cubic b-spline	Gaussian filter error rate	SSA error rate	Cubic b-spline error rate
d_fine	*Ra*(µm)	0.12622	0.12622	0.12527	0.12685	0	0.76%	0.50%
	*Rq*(µm)	0.15896	0.16043	0.15927	0.16107	0.92%	0.20%	1.31%
	*Rku*	3.3264	3.54712	3.55471	3.50192	6.63%	6.42%	5.01%
Polish	*Ra*(µm)	0.06161	0.06161	0.06091	0.06233	0	1.15%	1.16%
	*Rq*(µm)	0.08702	0.08768	0.08689	0.08854	0.76%	0.15%	1.72%
	*Rku*	10.45915	10.41407	10.36135	9.46133	0.43%	0.94%	10.55%
Mill	*Ra*(µm)	0.16423	0.16424	0.15389	0.16531	0.00%	6.71%	0.65%
	*Rq*(µm)	0.20113	0.20134	0.18819	0.20022	0.10%	6.87%	0.45%
	*Rku*	2.40393	2.48783	2.47912	2.46602	3.49%	3.03%	2.58%

The experimental results indicate that, compared with the ISO-recommended Cubic B-spline wavelet, SSA produces a filtering effect that is more consistent with the Gaussian filter. Moreover, the roughness parameter errors obtained by SSA remain within a reasonable range, thereby confirming its practical applicability.Considering that, in surface roughness measurement, data acquisition from the material surface is typically the most time-consuming step and that roughness signal separation is performed after acquisition, the requirement for real-time data processing is relatively low. Since metrological work places greater emphasis on accuracy and reliability, we consider it entirely acceptable to allow for a few additional seconds of computation time in exchange for higher-quality signal separation.

**Fig 7:**
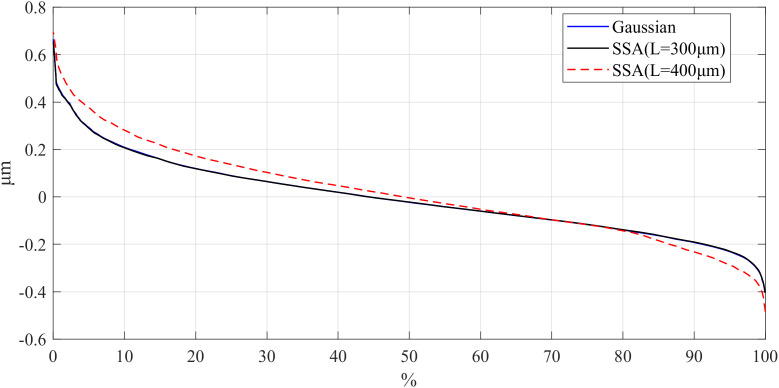
Comparison of BAC for different window lengths of parts: d_fine.

**Fig 8:**
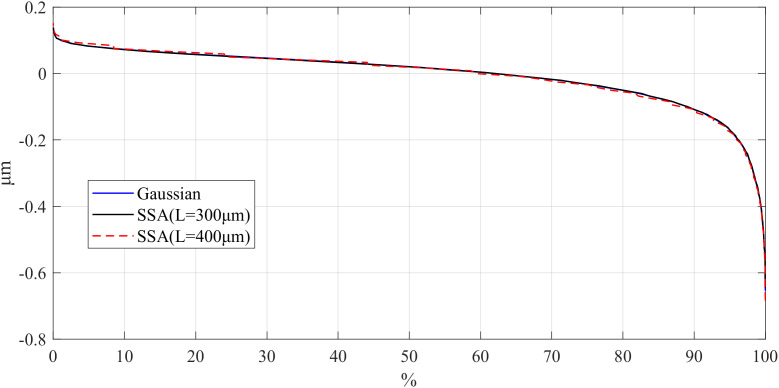
Comparison of BAC for different window lengths of parts: Polish.

**Fig 9:**
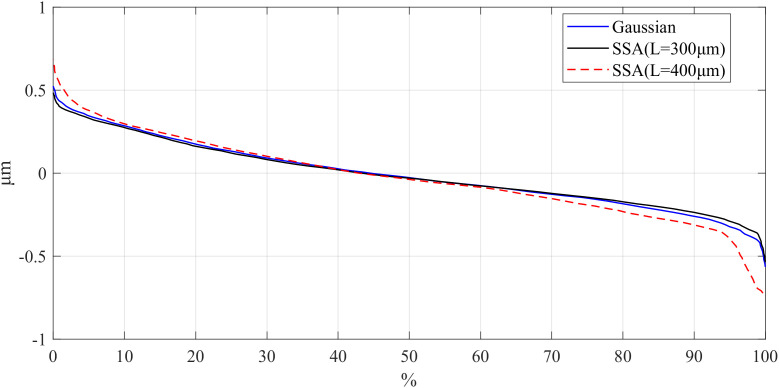
Comparison of BAC for different window lengths of parts: Mill.

On the other hand, the Bearing Area Curve (BAC) for the workpiece was obtained with different window length using the SSA, and the results are shown in Figs 7–9. BAC is a graphical representation based on surface height data, reflecting the material ratio of the surface at different heights. In short, it shows the percentage of material area in contact with a plane as it gradually descends from the highest point to the lowest point on the surface. The extremes of BAC curves are the most interesting to determine the sensibility of the filter applied in the peak and valleys zones. Different *L* values in SSA cause notable differences in BAC curve tails, reflecting variations in peak and valley preservation. Consequently, selecting an appropriate window length is essential for effectively utilizing SSA to filter the surface roughness.

### 4.2. Determination of the grouping method

**Fig 10:**
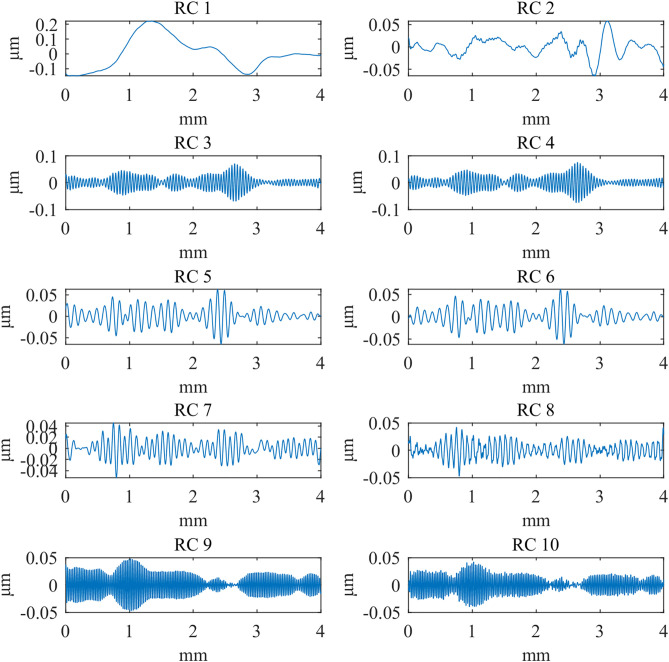
The first 10 reconstructed components: d_fine.

**Fig 11:**
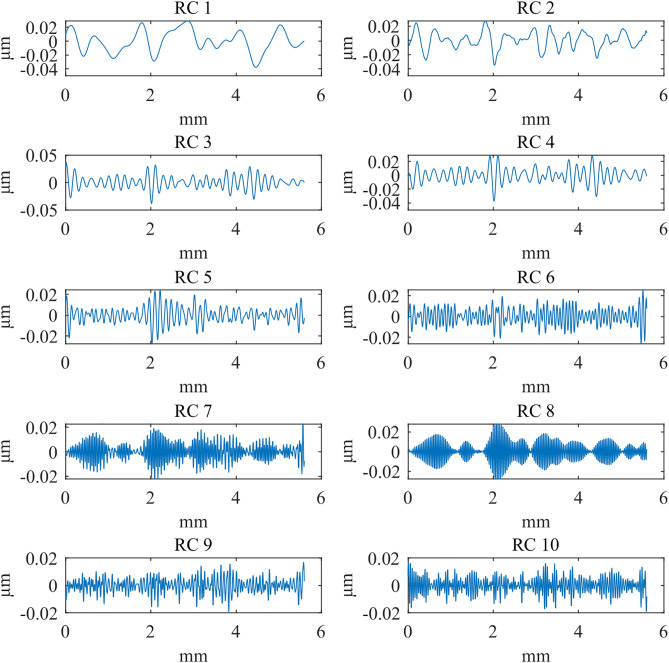
The first 10 reconstructed components: Polish.

**Fig 12:**
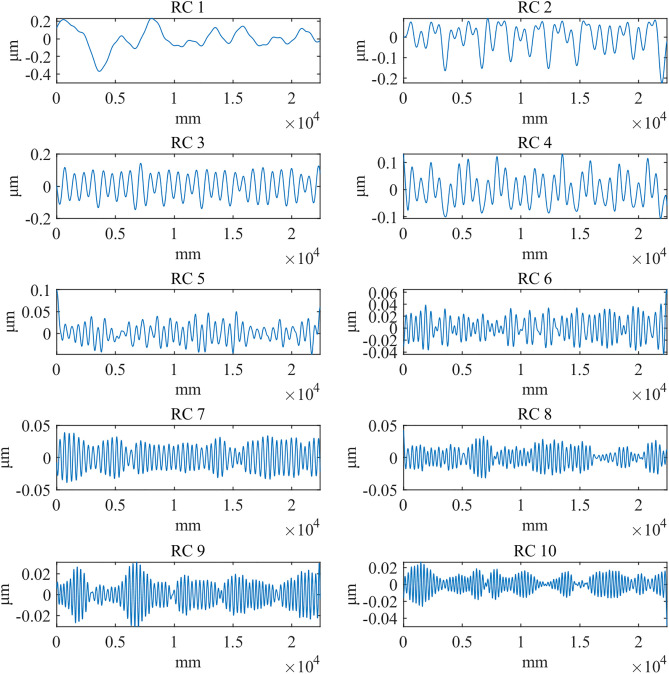
The first 10 reconstructed components: Mill.

In the context of the grouping method, Figs 10–12 presents the first ten reconstructed components when the window length (*L*) is set to 300μm. These components are sorted in descending order of singular value magnitude, reflecting decreasing frequency contributions. Upon evaluation, it was determined that the reconstructed component 1 closely corresponds to the low-frequency signal associated with the shape and waviness. In contrast, reconstructed component 2 and the subsequent components represent high-frequency signals related to the roughness. Therefore, the aggregate of reconstructed component 2 and the subsequent components is designated as the roughness signal.

**Fig 13:**
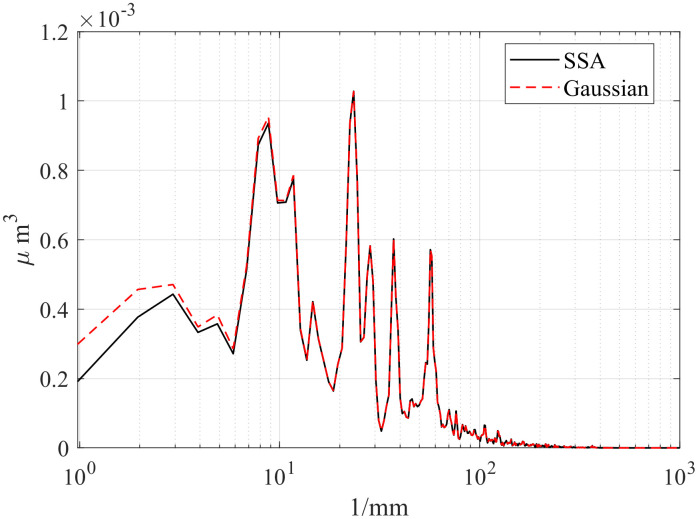
PSD plot at *L* = 300μm: d_fine.

**Fig 14:**
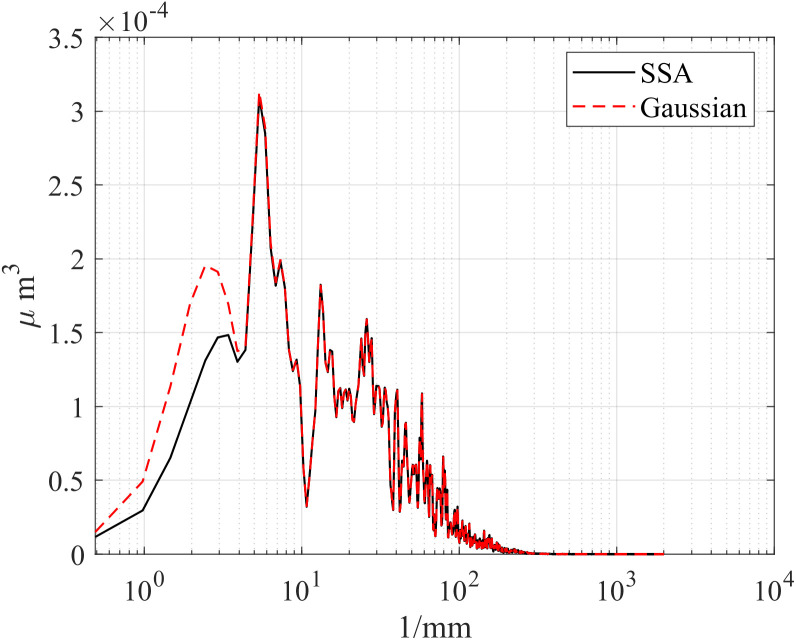
PSD plot at *L* = 300μm: Polish.

**Fig 15:**
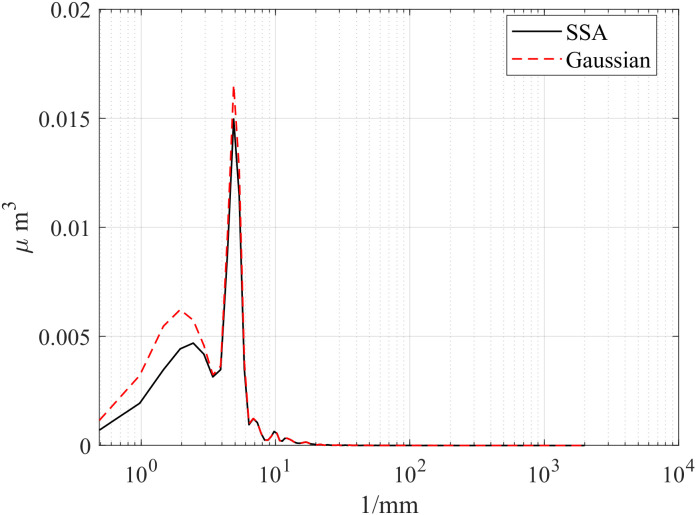
PSD plot at *L* = 300μm: Mill.

To compare SSA with the ISO 16610–21 Gaussian filter, we calculated the power spectral density (PSD) to characterize the energy distribution of the signal across different frequencies. Figs 13–15 presents the PSD results of SSA and the Gaussian filter for the d_fine, Polish, and Mill datasets, highlighting differences in the roughness spectral components. In the low-frequency range, a clear discrepancy is observed: SSA removes more low-frequency components, achieving a sharper separation of the signal and demonstrating superior performance compared with the ISO standard. This difference may stem from the smoothing nature of the Gaussian filter, which tends to ignore wavelength information outside its passband during bandwidth analysis. In contrast, SSA, as a non-smoothing and non-parametric method, can decompose a time series into statistically independent components (such as trend and harmonic terms) and reconstruct the signal without any prior assumption about its frequency content. As a result, SSA provides a more effective separation of waviness and roughness while better suppressing noise components.

We consider this a significant advantage. In our experiments, SSA demonstrated superior frequency separation compared to the ISO standard Gaussian filter, particularly in the transition region between waviness and roughness. This enhanced separation improves signal clarity and preserves features more effectively, increasing the accuracy of surface quality assessment. In industrial metrology, this advantage is crucial, as SSA allows for more precise differentiation of waviness and roughness on workpiece surfaces. By reconstructing surface profiles without relying on predefined frequency cutoffs, SSA provides a more comprehensive quantitative analysis, improving diagnostic capabilities in process monitoring, identifying root causes of surface anomalies, and ultimately contributing to better quality control in precision manufacturing.

## 5. Conclusions

This study systematically investigates the application of Singular Spectrum Analysis (SSA) in surface roughness measurement and provides a comparative analysis with the Gaussian filtering method specified in the ISO standard. Experimental results demonstrate that SSA can effectively isolate surface roughness signals and, with an appropriately selected window length, yield roughness parameter values comparable to those obtained using the Gaussian filter. Moreover, SSA retains less frequency content in the low-frequency range, enabling more precise differentiation between waviness and noise components within the roughness distribution. This characteristic highlights SSA’s advantages in enhancing frequency separation precision, improving feature preservation, and increasing the reliability of complex surface analysis, thereby ensuring high-accuracy measurement of intricate geometries and providing a new technical approach for functional feature extraction, process monitoring diagnostics, and root cause identification of surface anomalies. Therefore, SSA is not only an effective alternative to Gaussian filtering in surface roughness measurement but also demonstrates significant potential for broader applications in metrology and quality control.

Although this study has achieved certain results in applying SSA to the field of surface metrology, there are still some limitations:

1. Dependence on empirical grouping: The current component grouping approach primarily relies on manual, experience-based judgments and lacks a more objective, reasonable, and automated grouping strategy. This dependence may affect the stability and repeatability of the method under different signal characteristics or measurement conditions.
2. Insufficient dimensional extension: The present work focuses solely on one-dimensional roughness signals and has not yet extended the SSA method to the processing and evaluation of two-dimensional surface roughness data. Given that two-dimensional surface topography analysis is more widely used in engineering and manufacturing, future research should explore and validate the applicability and advantages of SSA in the two-dimensional context.

## Supporting information

S1 File NIST's measurement data for d_fine parts. (TXT)

S2 File The roughness data of d_fine parts separated by NIST. (TXT)

S3 File NIST's measurement data for Polish parts. (TXT)

S4 File The roughness data of Polish parts separated by NIST. (TXT)

S5 File NIST's measurement data for Mill parts. (TXT)

S6 File The roughness data of Mill parts separated by NIST. (TXT)
